# *Mycobacterium avium* subsp. *paratuberculosis* and microbiome profile of patients in a referral gastrointestinal diseases centre in the Sudan

**DOI:** 10.1371/journal.pone.0266533

**Published:** 2022-04-05

**Authors:** Wisal A. Elmagzoub, Sanaa M. Idris, Maha Isameldin, Nassir Arabi, Abdelmonem Abdo, Mustafa Ibrahim, Md Anik Ashfaq Khan, Franziska Tanneberger, Sahar M. Bakhiet, Julius B. Okuni, Lonzy Ojok, Ahmed A. Gameel, Ahmed Abd El Wahed, Michaël Bekaert, Mohamed E. Mukhtar, Ahmad Amanzada, Kamal H. Eltom, ElSagad Eltayeb

**Affiliations:** 1 Unit of Animal Health and Safety of Animal Products, Institute for Studies and Promotion of Animal Exports, University of Khartoum, Shambat, Khartoum North, Sudan; 2 Department of Biology and Biotechnology, College of Applied and Industrial Sciences, University of Bahri, Khartoum North, Sudan; 3 Faculty of Veterinary Medicine, Department of Pathology, University of Khartoum, Shambat, Khartoum North, Sudan; 4 Ibn Sina Specialised Hospital, Mohammed Najeeb St, Khartoum, Sudan; 5 Omdurman Islamic University, Omdurman, Sudan; 6 National centre for gastroenterology and liver diseases, Ministry of Health, Khartoum, Sudan; 7 Faculty of Veterinary Medicine, Institute of Animal Hygiene and Veterinary Public Health, University of Leipzig, Leipzig, Germany; 8 Department of Molecular biology, Institute of Endemic diseases, Khartoum, Sudan; 9 College of Veterinary Medicine, Animal Resources and Biosecurity (COVAB), Makerere University, Kampala, Uganda; 10 Faculty of Medicine, Department of Pathology, Gulu University, Gulu, Uganda; 11 Faculty of Natural Sciences, Institute of Aquaculture, University of Stirling, Stirling, Scotland, United Kingdom; 12 Faculty of Agriculture, Department of Agricultural Extension and Rural Development, University of Khartoum, Shambat Khartoum North, Sudan; 13 Department of Gastroenterology and Gastrointestinal Oncology, University Medical Centre Göttingen, Göttingen, Germany; 14 Faculty of Medicine, Al Neelain University, Khartoum, Sudan; Universita degli Studi di Sassari, ITALY

## Abstract

*Mycobacterium avium* subsp. *paratuberculosis* (MAP) causes Johne’s disease in animals with zoonotic potential; it has been linked to many chronic diseases in humans, especially gastrointestinal diseases (GID). MAP has been extensively studied in Europe and America, but little reports were published from Africa. Sudan is a unique country with close contact between humans and livestock. Despite such interaction, the one health concept is neglected in dealing with cases of humans with GID. In this study, patients admitted to the reference GID hospital in the Sudan over a period of 8 months were screened for presence of MAP in their faeces or colonic biopsies. A total of 86 patients were recruited for this study, but only 67 were screened for MAP, as 19 did not provide the necessary samples for analysis. Both real-time PCR and culture were used to detect MAP in the collected samples and the microbial diversity in patients´ faecal samples was investigated using 16S rDNA nanopore sequencing. In total, 27 (40.3%) patients were MAP positive: they were 15 males and 12 females, of ages between 21 and 80 years. Logistic regression analysis revealed no statistical significance for all tested variables in MAP positive patients (occupation, gender, contact with animal, milk consumption, chronic disease, etc.). A unique microbiome profile of MAP-positive patients in comparison to MAP-negative was found. These findings suggest that a considerable proportion of the population could be MAP infected or carriers. Therefore, increase awareness at community level is urgently needed to decrease the risk of MAP at human/animal interface. This study represents the first report of MAP in humans in the Sudan; nevertheless, a better view of the situation of MAP in humans in the country requires a larger study including patients with other conditions.

## 1. Introduction

*Mycobacterium avium* subsp. *paratuberculosis* (MAP) is the aetiological agent of paratuberculosis (Ptb) or Johne’s disease (JD)- a chronic intestinal infection in ruminants [1]. MAP can survive in soil and water for months or years [2] and resists heat treatment processes and, consequently, it is highly challenging to eliminate MAP contamination; even more, chlorination and filtration promote its growth [3]. Faecal materials of infected animals deliver contamination to milk and pastures [4]; so, MAP is possibly transmitted to humans through milk, dairy products and water supply systems as well as aerosols [5]. In addition, meat could be source of infection as a result of dissemination of infection or contamination by faeces during slaughtering [6].

For a long time, the zoonotic nature of MAP had been speculated because of the similarity between JD and an intestinal disease in human that later has been named Crohn’s disease (CD) [7]. Experimental infections in goats and cows with MAP isolated from CD patients supported this link [8,9]. Further studies investigating the causality of MAP and CD isolated MAP from milk and blood samples, from intestinal sections and biopsies [4,10] and also detected MAP nucleic acid by polymerase chain reaction (PCR) in blood and tissue biopsies from CD patients [10,11]. However, detection and isolation of MAP from the control groups (ulcerative colitis patients or non- inflammatory bowel disease (IBD)) in these studies indicated that MAP could be involved in many other diseases. Studies investigating MAP by PCR in faecal samples revealed that many individuals, healthy or with disease conditions other than IBD, could also be MAP carriers [12,13]. Therapeutic trials with antimycobacterial agents provided more evidences to the zoonotic potential of MAP [14]. MAP was postulated to have a role in development or progression of Human Immuno- Deficiency Virus infection, cardiovascular diseases, asthma and autism based on the immune dysfunction hypotheses [5,15,16].

The immunomodulatory abilities of mycobacteria have been extensively studied [17–21]. The heat-killed non-tuberculous mycobacteria were early found to exert immunomodulatory effects [22,23]. The immune response is stimulated either by the intact mycobacterial cell, synthetic analogues of bacterial structural components or the cellular breakdown products [17,24,25]. These immune triggers are associated with gut inflammation [24]. For example, an inflammatory response can be elicited through intestinal epithelial surface receptors as a result of activation of the NF-KappaB by bacterial lipopolysaccharides [26]. It has been revealed that the MAP N-glycolyl muramyl dipeptide is more potent than the N- acetyl muramyl dipeptide in inducing an immune reaction [17]. Moreover the genetic susceptibility to the infection supported the link of MAP with CD and other human diseases, such as type 1 diabetes mellitus (T1DM), rheumatoid arthritis, multiple sclerosis (MS), where the polymorphism in the SCL11A1 gene associated with these autoimmune conditions is similar to that associated with paratuberculosis (MAP infection in animals) [3]. Also, MAP have been associated with autoimmune conditions like T1DM, MS, sarcoidosis and thyroiditis based on the molecular mimicry with the mycobacterial heat shock proteins (HSPs). The molecular mimicry of two MAP proteins: HSP65 with the pancreatic protein, glutamic acid decarboxylase and MAP3865c with Znt8 predisposes to T1DM [3,7]. Furthermore, high antibody response had been reported against insulin epitopes and its’ MAP homologue in children with T1DM [3]. MAP3865c protein also predisposes to autoimmune thyroiditis [3]. Similarly, HSP65 was implicated in biliary cirrhosis and rheumatoid arthritis [3]. The immune response against the MAP also may lead to tissue damage, neuronal dysfunction and development of MS. In animal model, a booster dose with a vaccine containing the mycobacterial HSP65 gene could promote protection against MS and increased level of antibodies to HSP70 in Sardinian patients with MS and MAP DNA was detected in the peripheral blood in MS patients [27]. Later studies on experimental autoimmune encephalitis (EAE) using animal models have highlighted the potential of MAP to induce and exacerbate EAE [28]. Furthermore, orally administered MAP was found to stimulate immune response through the intestine affecting the gut-brain axis and modulating the inflammatory pathways and the synthesis of cytokines involved in the pathogenesis of EAE [29].

These diseases affect millions of people worldwide and mostly have no known cure to date pointing out MAP as possible public health threat. This diversity of association of MAP with human diseases is probably linked to the microbiome.

Microbiome has become a very important part of most studies, especially those related to the gastrointestinal tract (GIT). Microbiome was found to guide the development of the immune system, gut epithelium and brain. Many factors like age, diet and infectious pathogens, do affect the composition of the intestinal microbiome [30]. Intestinal dysbiosis, which is the alteration of microbial community, had been linked to immune-mediated conditions like rheumatoid arthritis, and many gastrointestinal illnesses including IBD. Moreover, MAP infected cattle were found to have different microbiome than MAP negative [31].

MAP in humans in the Sudan has never been investigated, neither in diseases that have been linked to it earlier (CD or IBD) nor in other diseases, more likely due to the scarce information on these diseases [32–34]. However, because of the relatively high prevalence of MAP in cattle in Khartoum, Sudan [35] in addition to the close contact between humans and animals, we hypothesize that MAP prevalence in human is higher than expected. This necessitates investigating this organism in humans as well. Moreover, no study has identified the microbial diversity in Sudanese patients suffering from GIT complaints.

**Fig 1:**
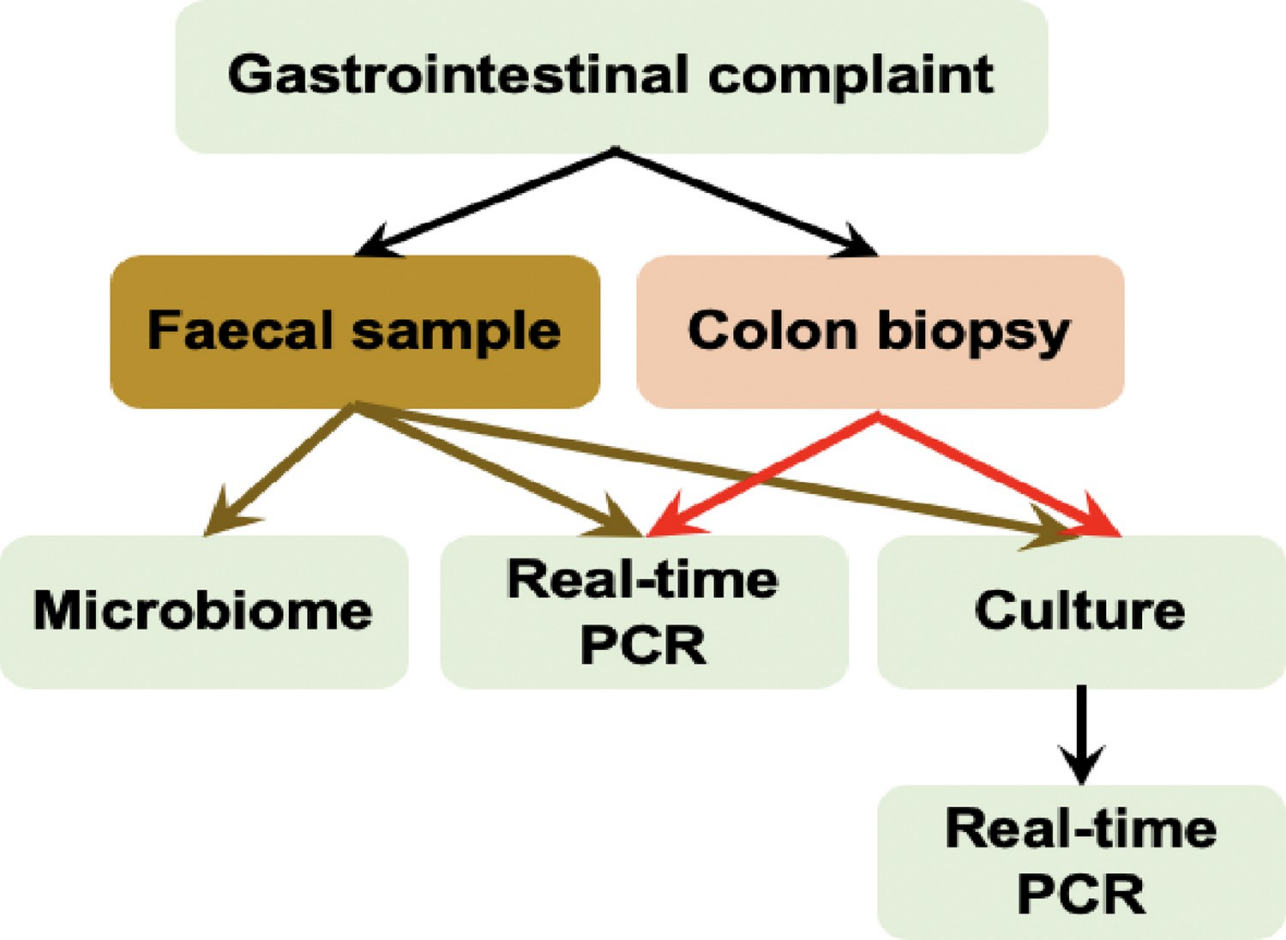
Study layout.

In this study, we investigated the presence of MAP by culture and real-time PCR in faecal and tissue samples of patients presented at a gastroenterology centre and its relation to patients’ demographic data as well as possible risk factors. The microbiota diversity between MAP positive and negative subjects were mapped to provide a better understanding of the diseases. Study layout is illustrated in “Fig 1”.

## 2. Materials and methods

### 2.1. Ethics statement

This study was approved by the IRB of Ibn Sina Specialized Hospital (ISSH) (ID:18022018) and IRB of University Medical Centre Göttingen, Germany (ID: 27/7/16). Written informed consents were obtained from all study participants. Samples were collected as part of the routine diagnosis and patient handling; and only leftover samples were used for the study. All samples were anonymized.

### 2.2. Study design and eligibility criteria

This is a cross sectional hospital-based study, carried out in ISSH, Khartoum, Sudan, a tertiary hospital to which considerable number of patients with gastrointestinal (GI) diseases are referred. The total number of patients who visited the GI Centre at ISSH during the period between July 2019 and Feb. 2020 were 86; they were all recruited as a total coverage convenient sample. However, eligible patients were those who agreed to participate in the study, consented on the use of the leftover of their diagnostic samples (faeces, colonic tissue biopsy) and were able to submit the samples. From each patient, data including age, gender, clinical history and symptoms, comorbidity, etc., were obtained (Supplementary file #1).

### 2.3. Molecular detection of MAP

Stool samples were processed for DNA extraction using QIAamp DNA Blood Mini Kit (Qiagen, Hilden, Germany) following the manufacturer’s instructions. The DNA was stored at -20°C until being used in real-time PCR.

A real time PCR assay described before [36] targeting the IS900 of MAP was used in this study. Briefly, the assay was performed in T- Optical Real-Time qPCR Thermal Cycler (Biometra, Analytik Jena, Jena, Germany) using a reaction mixture containing 10 μL *Taq* PCR Master mix (Qiagen), 0.5 μM of each primer (FP: 5´-TACCGCGGCGAAGGCAAGAC-3´ and RP: 5´-CGGAACGTCGGCTGGTCAGG-3´), 1 μM of the probe (5´-FAM-ATGACATCGCAGTCGAGCTG-BHQ-1-3´), 3 μL molecular biology grade water and 5 μL of the DNA template. The thermal cycler was programmed as follows: 10 min pre-incubation at 95°C followed by 40 cycles of 95°C for 15 s, 60°C for 30 s and 72°C for 35 s.

### 2.4. Culture methods for isolation of *Mycobacterium avium* subsp. *paratuberculosis*

All faecal and tissue samples were cultured for isolation of MAP. The samples were decontaminated using sterile solution of 0.75% hexadecyl pyridinium chloride (HPC) following standard decontamination protocols before being cultured in Middlebrook 7H11 agar (Sigma-Aldrich, Taufkirschen, Germany) slants supplemented with 0.6 mL, 50.0 g, 20.0 g, 0.03 g/L oleic acid-albumin-dextrose-catalase (OADC) and 2 mg/L of mycobactin J (Allied Monitor, USA). The cultured slants were incubated at 37°C for 4 weeks and then checked for growth every month for up to 20 months [37]. The growth of MAP was confirmed by real-time PCR. For DNA extraction, colonies suspension in Tris-EDETA buffer (pH 8.0) was prepared followed by heating at 99°C for 20 min with intermittent shaking. The real-time PCR was performed as described above.

### 2.5. Faecal microbiome

DNA was extracted from 100 mg faecal sample using the MagMax Microbiome Ultra Nucleic Acid Isolation kit (Applied Biosystems, USA) as described in the manufacturer’s instructions. In the tube provided with the kit containing beating beads, 100 mg faecal sample and 800 μL of the lysis buffer were added and mixed using Intelli-Mixer’s™ (ELMI, Riga, Latvia) at room temperature for 30 min, followed by centrifugation at 14000x *g* for 2 min. The DNA in the supernatant was purified using the binding beads solution prepared using the kits’ chemicals, followed by washing steps with the washing buffer and 80% alcohol. The last step, the elution, included heating at 75°C for 5 min with the elution buffer followed by pelleting the binding beads against magnet rack and the clear solution contained the DNA. The DNA concentrations were measured using Qubit 4.0. fluorometer and Qubit dsDNA HS Assay kit (Thermo Fisher Scientific, Waltham, MA, USA). A total of 10 ng of DNA was used for sequencing.

DNA library was prepared using the 16S barcoding kit (SQK-RAB204) of Oxford Nanopore Technologies (Oxford, UK) as instructed in the kit protocol. This included amplification of the 16S rDNA gene using primers and PCR mix provided with the kit followed by pooling of PCR products after measuring the DNA concentration. The prepared DNA library was then loaded on MinION Flow cell 9.4.1 fitted on the new MinION- Mk1C device (Oxford Nanopore Technologies). The sequencing was initiated by the installed software in the device and the sequencing time was adjusted to 12 h. Passed FASTQ files of successful reads were uploaded by the desktop application of Epi2me to the online EPI2ME platform (https://epi2me.nanoporetech.com/). The workflow Fastq 16S 2026 (Metrichor Agent, Oxford Nanopore Technologies) was chosen for analysis of sequences with quality score 10, minimum length filter of 1500 bases and BLAST E-value of 0.01.

### 2.6. Statistical analysis

To test the relationship between the MAP (dependent variable) and some risk factors (the independent variables) including patient’s occupation, age, gender, bowel habit, fresh rectal bleeding (FRB), hypertension (HTN), diabetes mellitus (DM), chronic renal failure (CRF), inflammatory bowel disease history (HxIBD), recent antibiotic treatment, animal contact, smoking, snuff, living with animal, drinking milk, etc, (see Supplementary file #1). Binary logistic regression (Logistic Regression Model) was applied using SPSS Statistics for Windows, version x. 23 (SPSS Inc., Chicago, Ill., USA). To test whether the variation between the relative abundance of MAP+ and MAP- significant or not, according to the species of bacteria, analysis of variance (ANOVA) method was implemented using SAS V. 9.3 (SAS institute, NC, USA) To test the quality of the sequence data, diversity indices: alpha (richness), gamma (total diversity) and beta (overlap; [gamma/alpha]-1), were calculated with the R/vegan package v2.5–3 and in R v4.0.1 (R Foundation, Vienna, Austria).

## 3. Results

### 3.1. MAP positivity and risk factors

**Fig 2:**
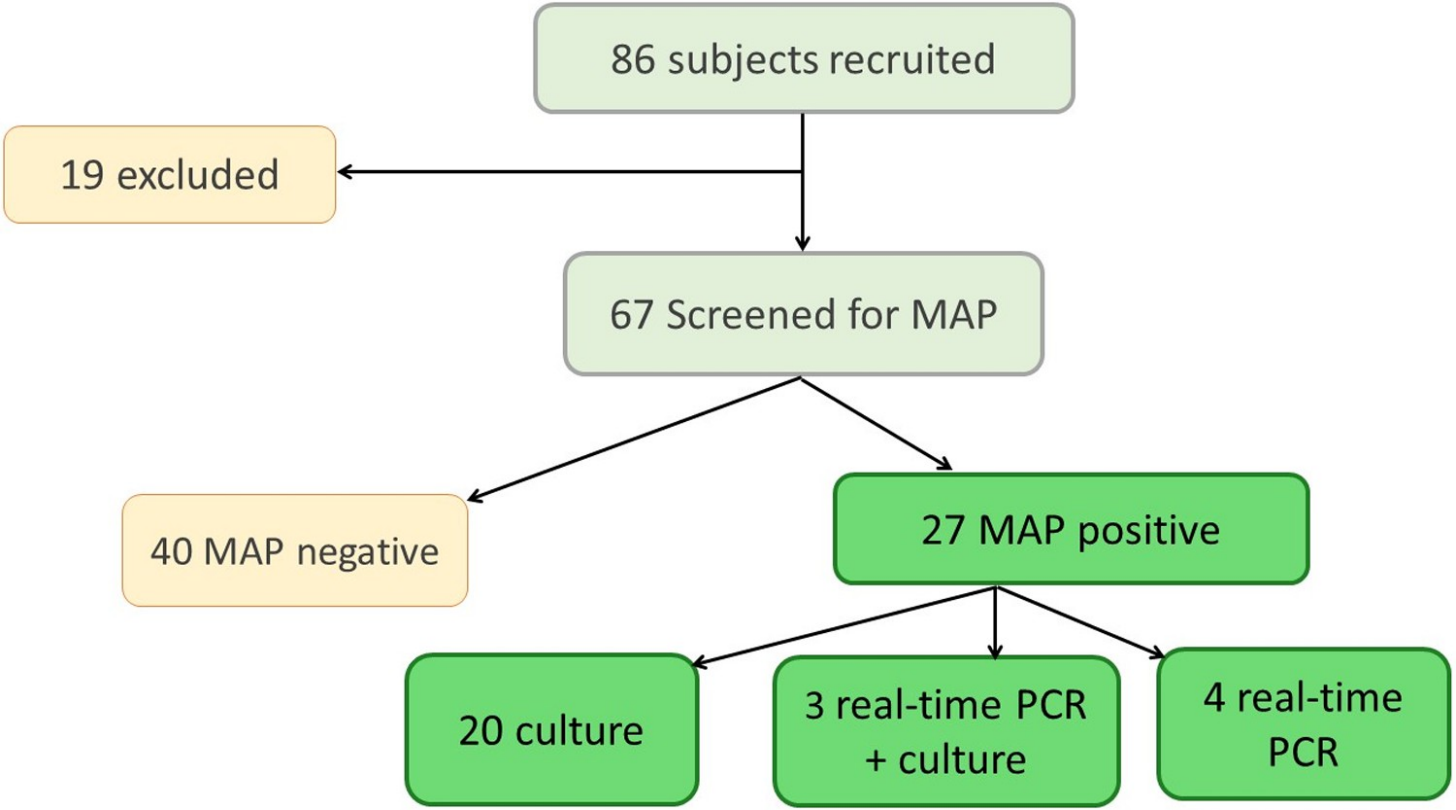
MAP positivity of tested samples of patients with gastrointestinal diseases.

In this study, 86 patients were recruited, from which 56.7% were males and 43.3% were females, within age range from 21 to 80 years. Samples (faecal or/and colonic biopsy) of 19 patients were not available and, therefore, were excluded from the analysis. In total, 13 colonic biopsies and 10 faecal samples were positive in real-time PCR after culture, 4 faecal samples were positive in direct real-time PCR and 3 faecal samples were positive in both direct real-time PCR and after culture “Table 1”. Upon this positivity rate (23/67, 40.3%), the samples were classified into two groups: MAP positive (MAP+) and MAP negative (MAP-) “Fig 2”.

**Table 1:** Positivity to *Mycobacterium avium* subsp. *paratuberculosis* in samples of recruited patients with gastrointestinal diseases in a GI centre Sudan (July 2019-Feb 2020).

Patient’s Code	Culture		Direct real-time PCR	
	Tissue	Faeces	Tissue	Faeces
R1	-	-	-	
R2	+	NP	-	NP
R3	-	+	-	+
R4	+	+	-	-
R5	-	-	-	-
R6	-	-	-	-
R7	-	NP	-	
R8	-	NP	-	
R9	-	NP	-	
R10	+	NP	-	NP
R11	-	-	-	-
R12	+	-	-	-
R13	-	-	-	
R14	+	-	-	-
R15	+	-	-	-
R16	-	NP	-	
R18	+	-	NP	
R20	-	+	-	-
R21	+	NP	NP	NP
R22	-	-	-	-
R23	-	-	-	-
R24	+	+	-	+
R25	NP	-	NP	
R26	+	-	-	NP
R27	-	NP	-	
R28	+	-	-	-
R29	-	NP	-	
R33	NP	-	NP	-
R34	NP	NP	NP	
R35	+	NP	-	NP
R36	-	NP	-	
R37	-	NP	-	
R38	NP	-	NP	+
R39	NP	-	NP	-
R40	NP	+	NP	-
R42	NP	-	NP	-
R43	NP	-	NP	+
R44	+	-	-	-
R46	NP	-	NP	-
R47	NP	-	NP	-
R48	NP	-	NP	-
R50	NP	+	NP	-
R51	NP	-	NP	-
R52	NP	-	NP	-
R53	NP	+	NP	-
R54	-	NP	-	NP
R55	NP	+	NP	-
R56	NP	+	NP	+
R57	NP	-	NP	-
R58	NP	-	NP	-
R59	NP	+	NP	-
R61	NP	-	NP	-
R62	NP	-	NP	-
R63	NP	-	NP	-
R64	NP	-	NP	+
R65	NP	-	NP	-
R70	-	NP	NP	NP
R73	NP	NP	NP	NP
R74	NP	-	NP	-
R76	-	NP	NP	NP
R78	-	NP	NP	NP
R82	-	NP	NP	NP
R83	-	NP	NP	NP
R87	NP	-	NP	-
R91	NP	-	NP	+
R92	-	NP	NP	NP
R93	-	NP	NP	NP

NP Not Provided, + positive,—negative.

Logistic regression “Table 2” of 15 variables showed insignificant (p>0.05) effect of all them on MAP+. However, diabetes mellitus, family history of inflammatory bowel disease, living with animal and snuff (Tobacco) increased the risk of MAP+.

**Table 2:** Logistic regression model of factors associated with positivity to *Mycobacterium avium* subsp. *paratuberculosis* (MAP+) in samples of patients with gastrointestinal diseases.

Variables	Odd ratio (OR)	S.E	P> z	95% CI
Age	1.009	0.020	0.650	0.970–1.050
Gender	0.599	0.717	0.474	0.147–2.438
Occupation	1.223	0.172	0.242	0.873–1.714
Change in bowel habit	1.147	0.763	0.857	0.257–5.114
Hypertension	0.410	0.776	0.250	0.090–1.875
Diabetes mellitus	2.271	0.800	0.305	0.473–10.898
Chronic renal failure	1.200	1.280	0.887	0.098–14.745
History of previous diagnosis of IBD	0.579	0.750	0.465	0.133–2.514
Family history of IBD	2.123	0.911	0.409	0.356–12.662
Recent antibiotic treatment	1.525	0.735	0.566	0.361–6.435
Animal contact	0.754	1.053	0.789	0.096–5.939
Living with animal	2.401	0.666	0.189	0.651–8.856
Drinking Milk	0.751	0.676	0.672	0.873–2.823
Smoking	0.775	0.824	0.757	0.154–3.894
Snuff	2.919	1.015	0.291	0.399–9.609

Pseudo R^2^ = 0.2: Hosmer–leweshow: Chi-square = 9.5; p > 0.05. IBD: Inflammatory bowel disease.

### 3.2. Microbial diversity in MAP positive patients

**Fig 3:**
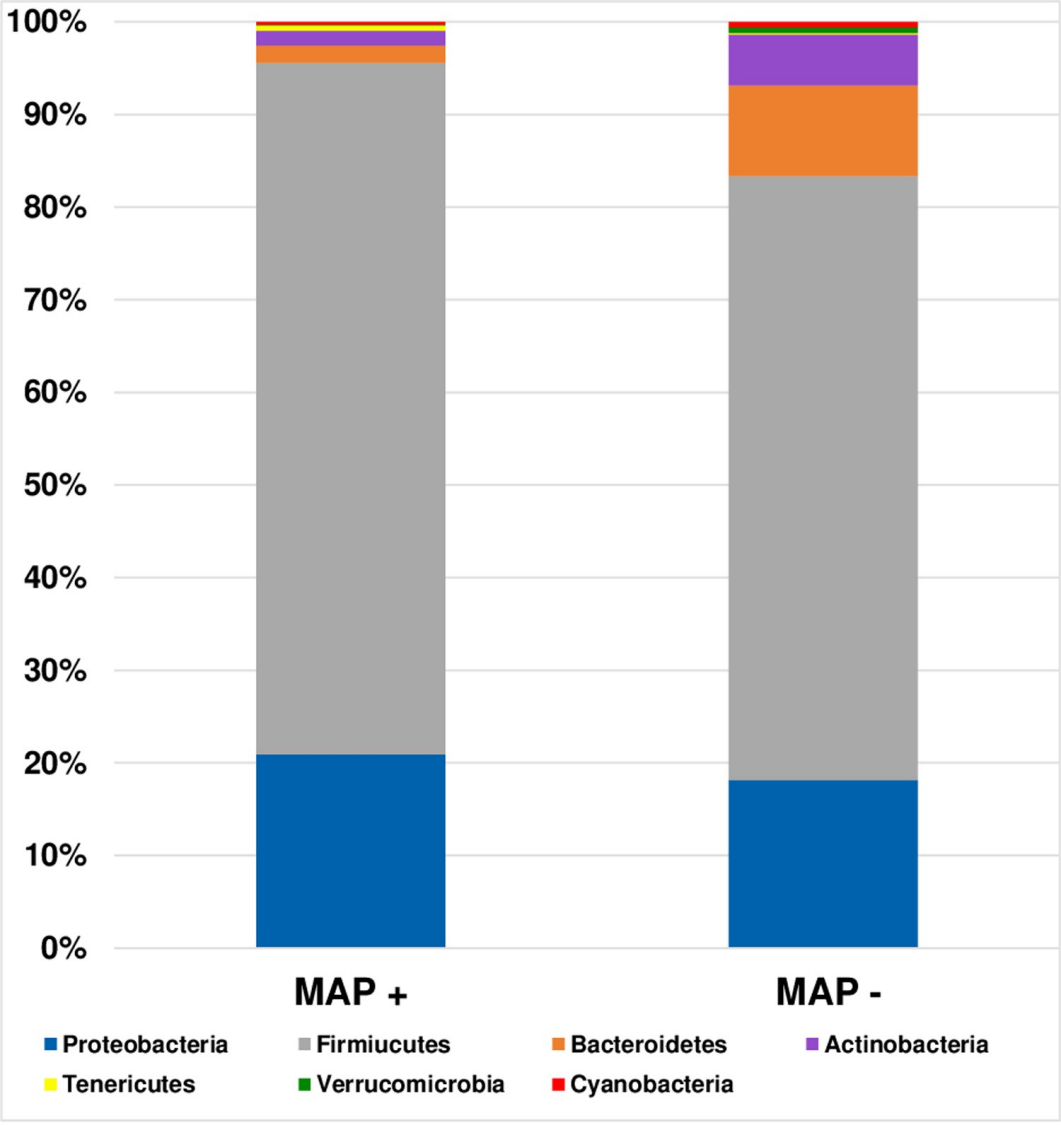
Phylum level faecal microbiome composition in MAP+ and MAP- groups.

**Fig 4:**
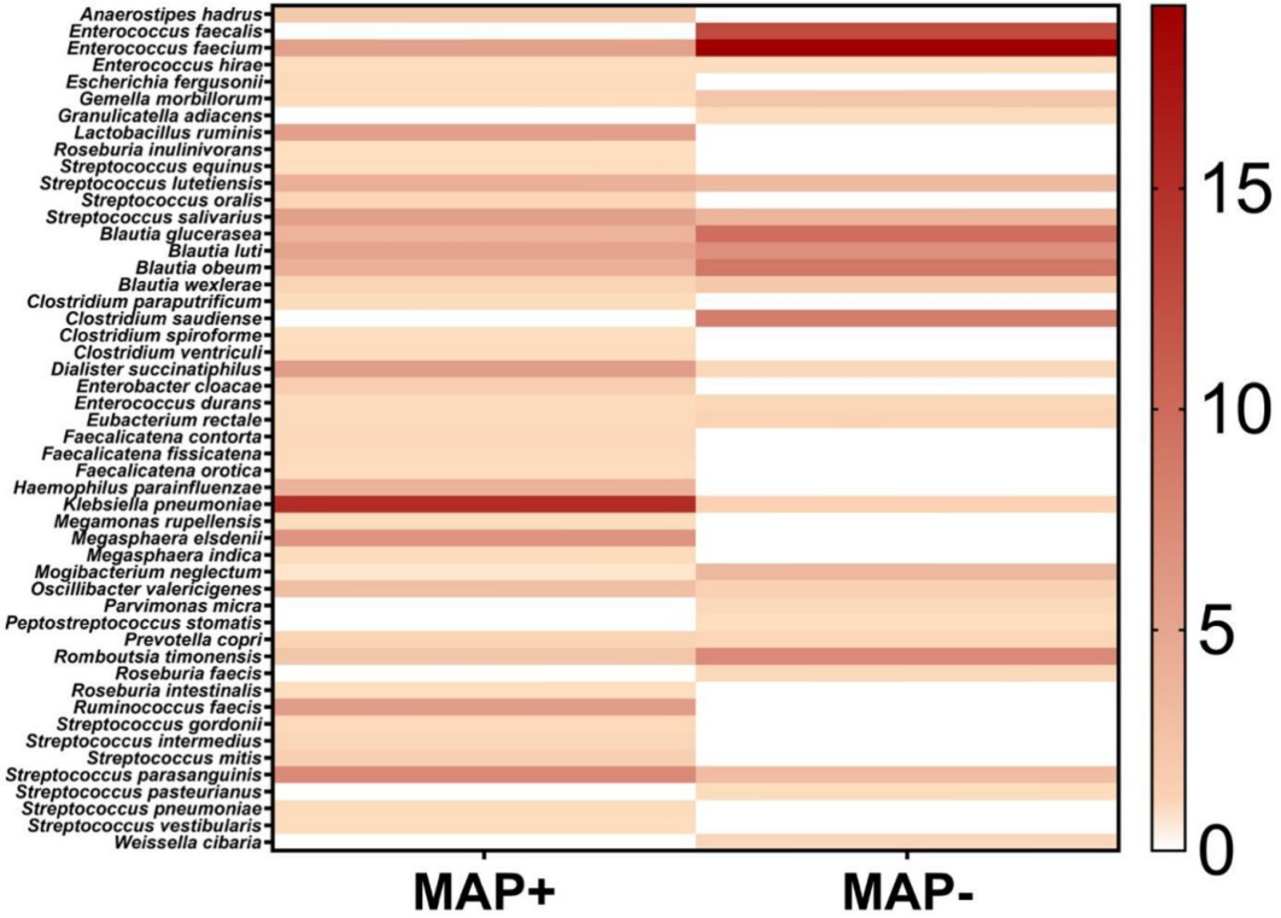
Heat map of the relative abundances of the most common species in the microbiome data of MAP+ and MAP- groups of patients with gastrointestinal diseases.

**Fig 5:**
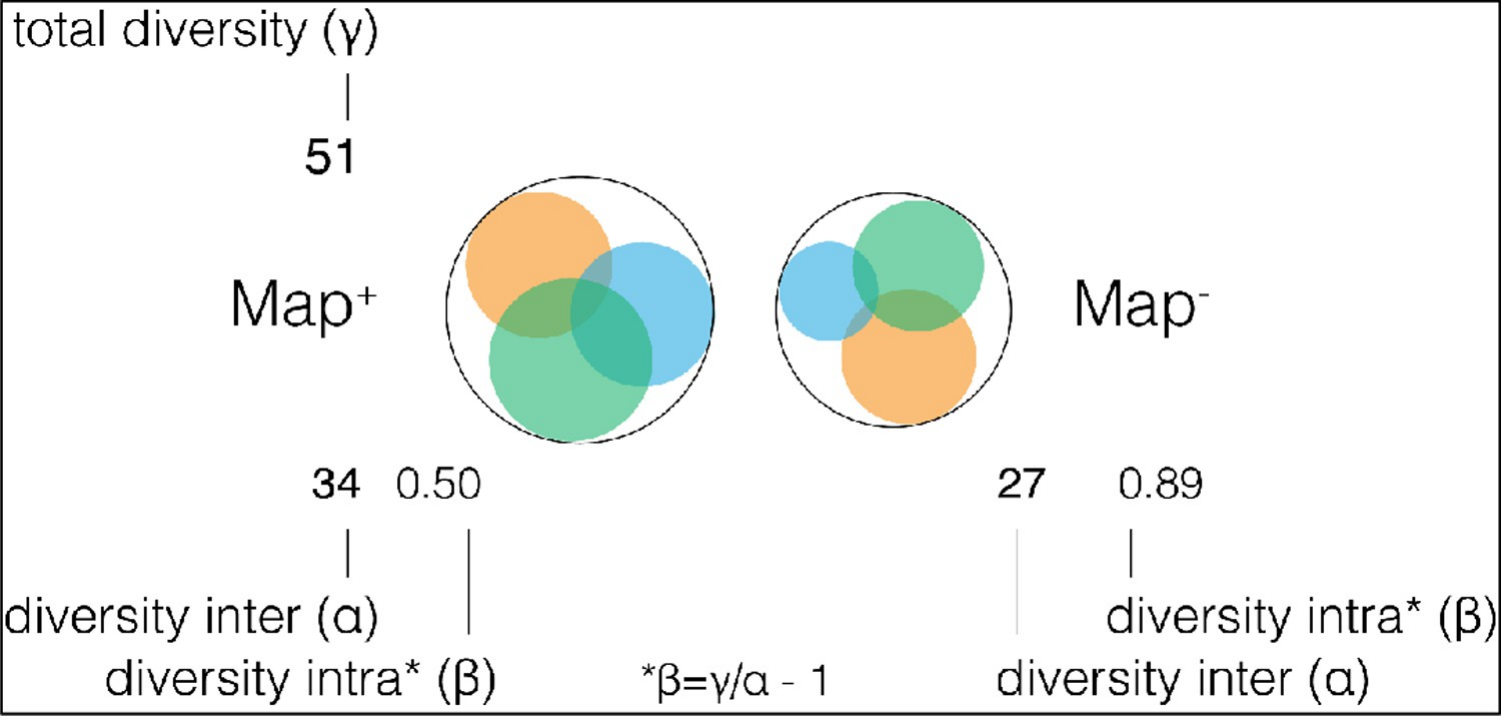
Diversity indices between MAP+ and MAP- samples. Alpha (richness), gamma (total diversity) and beta (overlap; [gamma/alpha]-1) were included.

A total of 44 out of 67 patients provided faecal samples for 16S rRNA nanopore sequencing. Despite the presence of enough nucleic acid, the quality score of sequences from 33 samples were not enough for data analysis. Raw sequencing data are available online: https://zenodo.org/record/5535630#.YVRhpi2B06g and BioSample accession: SAMN23097893 in NCBI BiSample Database. Sequences of 12 samples (7 MAP+ and 5 MAP-) were analysed and revealed variation at the phylum “Fig 3” and species “Fig 4” levels. *Dialister succinatiphilus* and *Klebsiella pneumoniae* were more abundant in the MAP+ group than MAP-. *Haemophilus parainfluenzae*, *Lactobacillus ruminis*, and *Ruminococcus faecis* were detected in the MAP+ group and absent in the other group. Among the MAP-, *Enterococcus faecium*, *Rombutsia timonensis*, *Mogibacterium neglectum*, and *Gamella morbillorum* were more abundant. The species of *Enterococcus faecalis*, and *Clostridium saudiense* were identified in MAP-, but absent in the MAP+. The *Blautia* species was found more abundant in MAP- group, while *Streptococcus* spp. was more in the MAP+ group. Two species were significantly (p<0.05) related to MAP+ patients: *Blautia wexlerae* (p<0.0325) and *Streptococcus lutetiensis* (p<0.0393) (Table 3). The bacterial composition varied greatly between samples, but overall, the MAP+ were more diverse than the MAP- (alpha-diversity, 34 and 27 respectively; beta-diversity 0.50, 0.89, respectively), “Fig 5”.

**Table 3:** Average abundance of bacterial species in faecal samples of patients with gastrointestinal problems grouped as negative or positive for *Mycobacterium avium* subsp. *paratuberculosis*.

Bacterial species	MAP+	MAP-
*Blautia glucerasea*	3.709a	9.678a
*Blautia luti*	5.168a	6.859a
*Blautia obeum*	4.019a	8.897a
*Blautia wexlerae*	1.111a	2.027b
*Dialister succinatiphilus*	5.690a	0.724a
*Enterococcus durans*	0.363a	0.847a
*Enterococcus faecium*	5.401a	19.144a
*Enterococcus hirae*	0.296a	0.335a
*Eubacterium rectale*	0.507a	1.101a
*Gemella morbillorum*	0.327a	2.356a
*Klebsiella pneumoniae*	15.244a	1.204a
*Mogibacterium neglectum*	0.078a	3.316a
*Oscillibacter valericigenes*	2.869a	1.207a
*Prevotella copri*	1.103a	0.831a
*Romboutsia timonensis*	2.325a	7.377a
*Streptococcus lutetiensis*	4.102a	3.261b
*Streptococcus parasanguinis*	7.343a	3.004a
*Streptococcus salivarius*	5.436a	3.672a

Figures with different letters are significantly different (p<0.05); MAP+: Positive for *Mycobacterium avium* subsp. *partuberculosis;* MAP-: Negative for *Mycobacterium avium* subsp. *paratuberculosis*

## 4. Discussion

For decades, *Mycobacterium avium* subsp. *paratuberculosis* has been debated as a cause of Crohn’s disease (CD); many investigations supported this view and also detected it in patients with many other conditions [8–11,37], pointing it out as zoonotic pathogen. However, as it is still more likely to be suspected in CD and other IBD diseases, this study aimed at investigating the presence of MAP in humans by targeting patients with chronic gastrointestinal diseases (GID), who have been referred to the GID reference hospital in the Sudan (ISSH). The positivity rate of MAP in the recruited eligible patients was about 40%. However, previous studies in other countries reported the detection of MAP in the faeces of individuals (target or control) with varying rates. In Germany [12], MAP DNA was detected by real-time PCR in the faeces of 2.09% (27/1293) of hospitalized patients suffering from diarrhoea. This lower rate of MAP+ German patients can be due to many factors: the target group (diarrhoeic patients only), while our study was focusing on GID patients; MAP was detected by real-time PCR only, which is less sensitive than culture, according to our results, in addition to differences in diet habits and possible susceptibility/ resistance to infection based on genetic variations. In Italy [38], MAP was detected in 9% (1/11) and 5.26% (1/19) of the faecal samples of the control and target (CD patients) groups, respectively, by a real- time PCR assay. Another study [39], also in Italy, documented a higher proportion of MAP+ faecal samples (48% of healthy individuals), although conventional PCR was used. However, in India [13], a lower rate of MAP+ faecal samples (2.9%, 3/101,) was reported by conventional PCR.

MAP was detected in some (7/27) subjects with type II diabetes mellitus (DM) in our study; although this was not significant in the Logistic Regression Model, the odd ratio (OR) shows that DM patients are 2-fold higher risk to be MAP+. Some investigations pointed out that MAP plays a role in the development of type I diabetes mellitus through molecular mimicry [7], but weak evidences were available for the connection of type II diabetes to MAP [5]. All patients in this study shared the GID complaints and abdominal disorders were one of the clinical conditions of MAP+ individuals in a previous study [13]. Also, MAP DNA in faeces was reported in conditions related to liver disorders [13], as in 2 cases in our study. These two patients were suffering ascites and jaundice, respectively, and both are indicators of liver disease.

Although not all of the MAP+ patients were living with or had animal contact, they shared the dietary routine of milk consumption. Infected animals usually excrete the organism in milk and faeces progressively [4] and with the thermal resistance of MAP [3], milk consumption is a potential risk factor for its transmission, especially with the high prevalence of MAP recorded recently [35]. MAP was also documented in apparently healthy individuals [13,39] and many other health disorders, including malaria, thyroid disorder, iron imbalance and anaemia [13]. As, numerous autoimmune diseases are thought to be developed as a result of molecular mimicry [7], these diseases might have not been yet diagnosed in such healthy individuals. Microbial triggers of chronic illnesses lead to disease development through interfering with immunoregulation directly due to the presence of the bacterium itself or its’ components and metabolites, or indirectly through inhibiting a vital function or process. MAP infects and multiplies within macrophages causing release of inflammatory mediators that predispose to local inflammation. On the other hand, microbial triggers could be the component of dead cell [2]. Therefore, MAP could infect humans but remains in latency in those who do not show overt symptoms of infection [40]. The fact that bacterial cell components and their derivatives trigger inflammatory pathways or autoimmune conditions explain why MAP may not be detected in all specimens [41] and contrarily it may be detected in genetically non-susceptible individuals [42–44]. Infected animals usually excrete MAP in their faeces and milk, even before developing disease symptoms or showing clinical signs. Although paratuberculosis has widely spread among cattle farms, milk and meat from these farms are not subjected to control; as a result, they contribute to human exposure [45]. Furthermore, the persistence of MAP in environment (soil, air) and water supply systems [45–47], render exposure to it as commonplace. However, negative results do not always exclude the presence of MAP.

Dysbiosis was linked to many chronic inflammatory diseases, e.g., *Bacteroidetes* were enriched in early onset cases of rheumatoid arthritis, and microbiome in IBD showed less diversity than healthy individuals [48]. Although the link of MAP to human diseases had been investigated extensively, no data on the possible association between MAP and change in microbiome is available yet. On the other hand, a few number of studies in animals investigated this association were conducted, but their results showed inconsistent change in microbiome pattern: in one study, MAP+ animals showed increased proportion of certain families belonging to the phyla *Bacteroidetes* and *Firmicutes* and reduced proportion of other families of *Firmicutes* and *Verrucomicrobia* phyla, while another study documented a link between MAP positivity and enrichment of certain families in the phyla *Actinobacteria*, as well as decreased abundance in families assigned to *Bacteroidetes*, and *Proteobacteria*, in contrast, others found an increase in abundance of *Proteobacteria* in MAP + animals and decreased abundance of Actinobacteria and Firmicutes [49]. In the present study, the MAP+ group showed higher abundance of the phyla *Proteobacteria* and *Firmicutes* compared to the MAP- group, which showed higher abundance of *Bacteroidetes* and *Actinobacteria*, similar change was observed in patients with irritable bowel syndrome [50]. In addition, some species were more abundant in MAP+ group. Nevertheless, *Blautia* spp. were notably diminished in MAP+ group. *Blautia* spp. are known to have potential probiotic function. The decreased abundance of *Blautia* spp. was found in patients with IBD, diabetes and colorectal cancer [51]. Moreover, this decrease in *Blautia* spp. was linked to metabolic inflammation and insulin resistance in obese individuals [51]. *Streptococcus* species were higher in the MAP+ group; the same was reported for IBD patients [52]. Despite these results, we cannot confirm that MAP alone had lead to these changes as other factors can affect the microbiome composition [30].

Two technical outcomes can be obtained from this study: first, culture was superior over the real-time PCR in the detection of MAP in patients´ samples; second, faecal microbiome from GID patients needs more standardization. Culture is the gold standard in bacteriology, but MAP cultivation is very difficult and requires long incubation time, which makes it difficult to use as surveillance method. Despite the high sensitivity of the real-time PCR, only few samples were positive in our study by molecular testing, while huge proportion were positive after culture. Therefore, applying real-time PCR can lead to underestimation of MAP situation in a particular population. In the microbiome study, all standard procedures (during collection, transportation, storage and handling) to produce sequences passing the quality score were followed [53]. Surprisingly, only 27.3% of sequenced samples had produced enough data. The difficulty in handling diseased *versus* healthy subjects must be considered in future studies. Possible solution is applying multiple sampling strategy within a given time frame.

The sample size was one of the limitations of the study: this was because: (1) the target group was patients with chronic GID complaints, who visited the GI centre for follow-up or were referred to the centre for a specific investigation and their follow-up was in a private clinic, (2) the collection period began in July 2019 and stopped in February 2020 following the lock down in response to the COIVD 19 Pandemic and after partial resumption of the GI centre activity, the patients were reluctant to visit the hospital in fear of getting COVID 19 infection (3) the time required for obtaining conclusive positive or negative results for MAP culture extended for months and recruitment of additional patients would have been impractical.

## 5. Conclusion

This is the first study investigating MAP in humans in the Sudan. The finding that more than 40% of the patients included in this study were MAP positive demonstrates that a considerable proportion of population could be MAP infected or carriers, which support our hypothesis that the prevalence of MAP in humans in the SUDAN is higher than would have been expected. In view of the high consumption rate of milk and dairy products in the Sudan and the considerable prevalence of MAP among dairy cattle, further investigations to estimate the bioburden of MAP in Sudanese people by involving patients with different conditions and healthy individuals are required to give more insight into its relation to other diseases and to get a clue on the actual situation of MAP. At the same time, such study would produce more accurate data on the faecal microbiome profile associated with MAP positivity.

Although the sample size in this study was not enough to draw a strong conclusion on the role of MAP in cases of GID complaints in the Sudan, it warrants further investigation in the future.

## Supporting information

S1 File (PDF)
