# Supplementary material for: Mycobacterium avium subsp. paratuberculosis and microbiome profile of patients in a referral gastrointestinal diseases centre in the Sudan
Source: PLoS One. 2022 Apr 5;17(4):e0266533. doi: 10.1371/journal.pone.0266533 (PMC8982859; doi:10.1371/journal.pone.0266533)
Supplement: S1 File — (PDF) [file pone.0266533.s001.pdf]

Date / / **Questionnaire of Sudanese patient's MAP with crohn's disease**

1. Name …………………………………………………………
2. File No. ☐
3. Contact mobile: 1-………………………………….2-…………………………………….3-……………………….
4. Occupation……………………
5. Age: ☐
6. Gender: M ☐ F ☐
7. Tribe ……………………………Origin…………………………………Resident……………………………………

History of presenting symptoms:

8. Change in bowel habit: yes ☐ No ☐ if yes, duration is…………………………….
9. Meleana: yes ☐ No ☐ if yes, duration is…………………………….
10. Fresh rectal Bleeding: yes ☐ No ☐
    a. If yes, duration is………………frequency…………amount…………….(1 cup of tea =150 ml)
    b. If yes, is it? Before defecation ☐ mixed with stool ☐ staining the stool ☐ after defecation ☐ without defecation ☐
11. Mucous discharge: yes ☐ No ☐ if yes, frequency…………..amount………….duration………
12. Anal pain: yes ☐ NO ☐ if yes, duration is…………………………….
13. Deep rectal pain: yes ☐ NO ☐ if yes, duration is…………………………….
14. Fecal/air incontinence: yes ☐ NO ☐ if yes, duration is…………………………….
15. Straining during defecation: yes ☐ NO ☐ if yes, duration is…………………………….
16. Tenesmus: yes ☐ NO ☐ if yes, duration is…………………………….
17. Pile: yes ☐ NO ☐ if yes, duration is…………………………….
18. Fistula: yes ☐ NO ☐ if yes, duration is……………………………
19. Loss of appetite: yes ☐ NO ☐ if yes, duration is………………………….
20. Loss of w.t: yes ☐ NO ☐ if yes, duration is…………………………….
21. Passing air or faeces per urethra or vagina? Yes ☐ No ☐
22. Abd. Pain: yes ☐ NO ☐ if yes, site…………nature…………… duration is………
    a. If yes, is it? colicky ☐ vague ☐ burning ☐ stabbing ☐
23. Vomiting: yes ☐ NO ☐ if yes the color is………………the duration………..
24. Jaundice: yes ☐ NO ☐ if yes, duration is…………………………………….
25. Abd. Distension: yes ☐ NO ☐ if yes, duration is……………………………….
26. Hemoptosis: yes ☐ NO ☐ if yes duration is……………………………….
27. HTN: yes ☐ NO ☐ if yes duration is…………………………treatment………….
28. DM: yes ☐ NO ☐ if yes duration is………………………treatment...............
29. CRF: yes ☐ NO ☐ if yes duration is………………………treatment...............
30. Other chronic illnesses ……………………………………….
31. History of IBD: yes ☐ No ☐ if yes type…………………………duration is…………………..
32. Recent antibiotic yes ☐ NO ☐ if yes,type……………. For how long…………………
33. When started treatment?...................
34. What is the treatment?..................
35. Any contact with MAP patient? yes ☐ NO ☐
36. Family history of similar condition: yes ☐ NO ☐ if yes degree of relation…………………..
37. Is he/she smoker? yes ☐ NO ☐

38. Is he/she alcoholic? yes ☐ NO ☐
39. Is he/she snuffer? yes ☐ NO ☐
40. Animal contact yes ☐ NO ☐ if yes, which animal…………..duration of contact……
41. Living with animal yes ☐ NO ☐
42. Milk drink? yes ☐ NO ☐
43. Contact with MAP Patient yes ☐ NO ☐ if yes, which animal………………duration of contact……….

Physical examination:

44. Palor : yes ☐ No ☐
45. Jaundice: Yes ☐ No ☐
46. Abdomen distended: yes ☐ No ☐
47. Abdominal mass: yes ☐ No ☐
48. Ascitis: yes ☐ No ☐
49. Splenomegally: yes ☐ No ☐
50. Hepatomegally: yes ☐ No ☐

Investigation:

51. ESR ☐
52. CRP ☐
53. CT abdomen: terminal ielum mass ☐ terminal ileum stricture ☐ LN ☐ ascites ☐ hepatic mets ☐ involvement of surrounding structure ☐ other……………………..
54. Colonoscopy finding:,,………………………………………….
